# Impact of Sugarcane–Pumpkin Intercropping on Soil Microbial Diversity

**DOI:** 10.3390/microorganisms13071703

**Published:** 2025-07-20

**Authors:** Xianglei Chen, Zhikui Cheng, Liwen Su, Xialei Huang, Yan Deng, Wenhui Bai, Zhihao Chen, Baoshan Chen, Peng Wang, Hongguang Pang, Zhengguo Liu

**Affiliations:** 1 College of Agriculture, Guangxi University, Nanning 530004, China; 2317391009@st.gxu.edu.cn (X.C.); 2217401017@st.gxu.edu.cn (Z.C.); 2317401015@st.gxu.edu.cn (L.S.); 2417302005@st.gxu.edu.cn (X.H.); 2217302002@st.gxu.edu.cn (Y.D.); 2217391001@st.gxu.edu.cn (W.B.); 2217391003@st.gxu.edu.cn (Z.C.); chenyaoj@gxu.edu.cn (B.C.); wangpeng@gxu.edu.cn (P.W.); 2 Department of Agriculture and Food Science, Shijiazhuang University, Shijiazhuang 050035, China; 3 Hebei Provincial International Joint Research Center for Green Agricultural Biological Agents, Shijiazhuang 050035, China

**Keywords:** intercropping, sugarcane, pumpkin, soil microbial community

## Abstract

Intercropping has been widely proven to boost agricultural yields and control diseases. This study examined the impact of sugarcane monoculture (SM) and sugarcane–pumpkin intercropping (IP) systems on soil physicochemical characteristics and microbial community dynamics. Compared to monoculture, intercropping significantly increased soil pH by 8.82% and total potassium (TK) by 17.92%, while reducing soil organic matter (SOM) by 25.56%. Bacterial communities under intercropping exhibited significantly higher alpha and beta diversity, whereas fungal community diversity remained unaffected. Notably, the relative abundances of certain taxa with known roles in plant growth promotion and pathogen suppression—such as *Anaeromyxobacter*, *Nitrospira*, and *Massilia*—were enriched. Canonical correlation analysis revealed that bacterial community composition was strongly associated with TK, while fungal community structure correlated with variations in soil available nitrogen (AN). These findings indicate that sugarcane–pumpkin intercropping reshapes soil microbial communities and contributes to some improvement in soil nutrient availability.

## 1. Introduction

Intercropping, the practice of cultivating multiple crops on the same land, enhances the utilization of environmental resources and improves biomass conversion efficiency [1]. Previous research has demonstrated that intercropping can alter rhizosphere microbial communities and soil physicochemical properties. For example, sugarcane–peanut intercropping modifies microbial abundance and enzyme activity, thereby regulating genes involved in nitrogen and phosphorus cycling as well as organic matter turnover [2]. Similarly, potato–onion intercropping has been shown to increase the alpha and beta diversity of both bacterial and fungal communities, along with bacterial abundance [3]. Moreover, increased microbial diversity under intercropping can enhance the nutrient uptake efficiency and mitigate the impact of soil-borne pathogens [4].

Sugarcane (*Saccharum officinarum* L.), a key global sugar crop, plays a vital role in agricultural economies and sugar supply chains worldwide [5,6]. Accounting for approximately 80% of global sugar production, sugarcane also serves as an essential raw material for animal feed, paper production, and ethanol manufacturing [7]. Its wide row spacing and extended growing period make it particularly suitable for intercropping with low-growing, short-season crops [8]. Pumpkin (*Cucurbita moschata*) is a commonly cultivated trailing plant with global distribution [9,10]. Pumpkins grow during the early to mid-growth stages of sugarcane, effectively utilizing sunlight and land resources that would otherwise remain idle in sugarcane fields during this early phase. Furthermore, sugarcane is deep-rooted while pumpkins are shallow-rooted. Intercropping pumpkins with sugarcane allows for efficient utilization of nutrients in the topsoil layer and minimizes competition for nutrients with the sugarcane [11,12].

Although various sugarcane intercropping systems have been studied, most have focused on legumes [13,14], while the effects of sugarcane–pumpkin intercropping on rhizosphere soil fertility and microecological conditions remain largely unexplored. To address this gap, field experiments were conducted with two treatments: sugarcane monoculture and sugarcane–pumpkin intercropping. Soil microbial community abundance and composition were assessed using quantitative PCR (qPCR) and Illumina sequencing. The study hypothesized that: (1) intercropping would improve soil quality relative to monoculture; (2) microbial diversity and abundance would be higher under intercropping than monoculture; and (3) intercropping could enhance plant growth and increase the abundance of certain microbes with the potential to suppress pathogen growth.

## 2. Materials and Methods

### 2.1. Field Plots and Experimental Design

The field experiment was conducted at Shajing Farm (22°48′ N, 108°12′ E) in Jiangnan District, Nanning City, Guangxi Province, China. The region experiences a subtropical monsoon climate, with mean annual temperature and precipitation recorded at 21.8 °C and 1286 mm, respectively. The soil type was sandy, with baseline fertility parameters as follows: available nitrogen at (AN) 68.74 mg kg^−1^, available phosphorus at (AP) 23.45 mg kg^−1^, available potassium at (AK) 70.13 mg kg^−1^, organic matter at 16.09 g kg^−1^, and a pH of 6.5.

The study utilized the sugarcane (*Saccharum officinarum* L.) cultivar ‘Zhongzhe 9′ and pumpkin (*Cucurbita moschata*) cultivar ‘Xiyang’. Two cropping systems were established: sugarcane monoculture and sugarcane–pumpkin intercropping. A randomized complete block design was implemented with three replicates per treatment, resulting in six plots (each measuring 6.0 m × 6.0 m). In both systems, five rows of sugarcane were planted with a row spacing of 1.3 m and an intra-row spacing of 0.1 m. In the intercropping treatment, pumpkins were interplanted between sugarcane rows spaced 1.5 m apart. The sugarcane was planted in early February 2024, and the pumpkin seedlings were transplanted in early March.

### 2.2. Soil Sampling

On 20 June 2024, rhizosphere soil samples were collected by randomly selecting nine sugarcane plants per treatment. The sugarcane plants were uprooted and gently shaken to dislodge loosely adhered soil for physicochemical analysis. Subsequently, the rhizosphere soil tightly attached to the roots was scraped off using sterile brushes into aseptic bags for soil microbial community studies. Every three plants were pooled to form one biological replicate, yielding three replicates per treatment. Each composite sample was divided into two portions: one part was flash-frozen in liquid nitrogen, transported to the laboratory, and stored at −80 °C for DNA extraction; the other was air-dried, sieved through a 2 mm mesh, and used for physicochemical analyses.

### 2.3. Measurement of Soil Properties

Soil properties were analyzed following standardized protocols [15]. Soil pH was measured potentiometrically in a 1:2.5 soil-to-water suspension. AN was quantified via the alkali-hydrolyzable diffusion method, while AP was extracted using sodium bicarbonate and analyzed by molybdenum–antimony colorimetry. AK was extracted with ammonium acetate and measured by flame photometry. Total potassium (TK) was measured using NaOH fusion coupled with flame photometry. Soil organic matter (SOM) content was measured by potassium dichromate oxidation with external heating.

### 2.4. Soil DNA Extraction, Amplification, and Sequencing

DNA was extracted from 0.25 g of fresh soil using the TIANamp Soil DNA Kit (Tiangen Biotech Co., Ltd., Beijing, China) following the manufacturer’s protocol. DNA integrity was assessed via 1% agarose gel electrophoresis, and concentrations were quantified using a UV spectrophotometer. The V3–V4 region of the bacterial 16S rRNA gene was amplified with primers 341F (5′-CCTAYGGGRBGCASCAG-3′) and 806R (5′-GGACTACNNGGGTATCTAAT-3′) [16], while the fungal ITS1-5F region was targeted using primers ITS5F (5′-GGAAGTAAAAGTCGTAACAAGG-3′) and ITS1R (5′-GCTGCGTTCTTCATCGATGC-3′) [17]. Each PCR reaction was conducted in a 15 µL volume containing Phusion High-Fidelity PCR Master Mix (New England Biolabs), 0.2 µM of each primer, and approximately 10 ng of template DNA. The thermal profile consisted of initial denaturation at 98 °C for 1 min, followed by 30 cycles of 98 °C for 10 s, 50 °C for 30 s, and 72 °C for 30 s, with a final extension at 72 °C for 5 min. Three technical replicates were performed per sample, with sterile water serving as a negative control to monitor potential contamination. PCR products were purified using magnetic bead-based methods and pooled in equimolar concentrations. After thorough mixing, target bands were detected, recovered, and used to construct sequencing libraries with index barcodes. Library quality was verified using Qubit fluorometry, real-time PCR, and bioanalyzer-based fragment analysis. Libraries were then pooled and sequenced on Illumina platforms based on library concentration and target data yield.

### 2.5. Sequencing Data Analysis

Paired-end reads were demultiplexed using unique barcode sequences and trimmed to remove barcode and primer regions. Merging of paired-end reads was performed using FLASH (v1.2.11; http://ccb.jhu.edu/software/FLASH/) (accessed on 20 July 2024), and optimized for overlapping reads from opposite ends of the same DNA fragment to generate raw tags [18]. Quality filtering of raw tags was conducted using fastp (v0.23.1) to obtain high-quality clean tags [19]. Tag sequences were aligned against reference databases—SILVA (16S/18S; https://www.arb-silva.de/) (accessed on 11 August 2024) and UNITE (ITS; https://unite.ut.ee/) (accessed on 11 August 2024)—to identify chimeric sequences. Chimera removal was performed using the VSEARCH package (v2.16.0; https://github.com/torognes/vsearch) (accessed on 16 August 2024), resulting in a set of high-quality effective tags [20].

Subsequent denoising and feature table generation were carried out with the DADA2 pipeline implemented in QIIME2, producing high-resolution amplicon sequence variants (ASVs) [21]. Taxonomic assignment of ASVs was conducted using the SILVA database (release 138.1) for bacterial sequences and the UNITE database (v9.0) for fungal sequences via the classify-sklearn algorithm in QIIME2 (v2022.2) [22,23].

### 2.6. Statistical Analysis

Alpha diversity indices of soil microbial communities were calculated using QIIME2. Soil physicochemical parameters, microbial alpha and beta diversity indices, and the relative abundances of bacterial and fungal taxa at the phylum and genus levels between monoculture and intercropping treatments were compared using Student’s *t*-test. Statistical analyses were conducted with SPSS (Version 27.0, Armonk, NY, USA). Venn diagrams were generated using the VennDiagram package in R (Version.3.2.0, Vienna, Austria). Non-metric multidimensional scaling (NMDS) analysis was performed in R with the ade4 and ggplot2 packages. Beta diversity was assessed based on taxonomic and phylogenetic metrics using weighted UniFrac distances. Linear discriminant analysis effect size (LEfSe) was employed to identify high-dimensional biomarkers differentiating the two treatments, applying a logarithmic LDA score threshold of 3.0 for discriminative features. Canonical correspondence analysis (CCA) and related visualizations were completed in R.

## 3. Results

### 3.1. Soil Physicochemical Properties

The soil physicochemical properties under the monoculture and intercropping systems are presented in Table 1. Compared to the sugarcane monoculture, the intercropping system significantly elevated soil pH and TK content, while substantially reducing SOM levels (*p* < 0.05). No significant differences were detected in AN, AP, or AK between the two treatments (*p* > 0.05).

### 3.2. Amplicon Sequencing Data and Shared OTUs

A total of 639,903 bacterial 16S rRNA and 671,633 fungal ITS sequences were obtained from six soil samples representing monoculture and intercropping systems. Following quality control procedures—including filtering, alignment, pre-clustering, and removal of chimeras and singletons—530,468 high-quality 16S rRNA sequences and 617,440 ITS sequences were retained.

**Figure 1:**
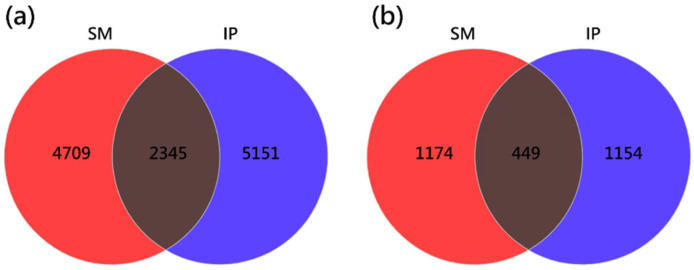
Venn plot of bacterial (**a**) and fungal (**b**) OTU quantity in soil samples. SM: sugarcane monoculture; IP: intercropping pumpkin.

Venn diagram analysis identified 12,205 bacterial and 3177 fungal ASVs across all samples (Figure 1). Among these, 2345 bacterial ASVs and 449 fungal ASVs were shared between the two cropping systems.

### 3.3. Soil Microbial Community Diversities and Structures

**Figure 2:**
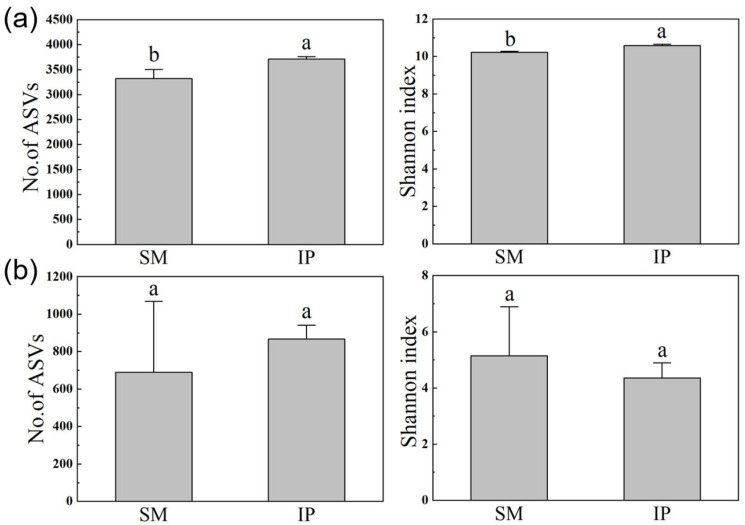
Amplicon Sequence Variant (ASV) richness and Shannon diversity index of bacterial (**a**) and fungal (**b**) communities under sugarcane monoculture (SM) and pumpkin intercropping (IP). Error bars indicate standard errors. Different letters denote significant differences (*p* < 0.05; Student’s *t*-test).

The intercropping system significantly increased both bacterial ASV richness and Shannon diversity compared to the monoculture (*p* < 0.05; Figure 2a), whereas fungal ASV richness and diversity showed no statistically significant differences between treatments (*p* > 0.05; Figure 2b).

**Figure 3:**
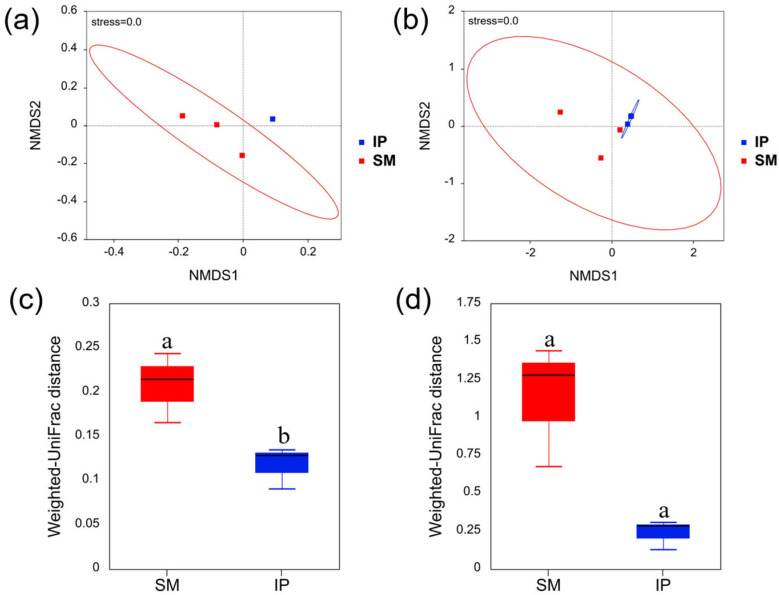
Beta diversity of bacterial (**a**,**c**) and fungal (**b**,**d**) communities under sugarcane monoculture (SM) and pumpkin intercropping (IP) based on weighted UniFrac distances. Different letters indicate significant differences (*p* < 0.05; Student’s *t*-test).

The NMDS analysis showed that the stress values of bacterial and fungal communities were both 0.00 (Figure 3a,b), indicating that NMDS can accurately reflect the degree of variation between groups. The NMDS plots showed a distinct separation of bacterial communities between monoculture and intercropping treatments (Figure 3a), while fungal communities exhibited partial overlap (Figure 3b). Beta diversity analysis based on weighted UniFrac distances confirmed that intercropping significantly altered the structure of soil bacterial communities (*p* < 0.05; Figure 3c), whereas no significant differences were observed in fungal communities (*p* > 0.05; Figure 3d).

### 3.4. Soil Bacterial Community Composition

**Figure 4:**
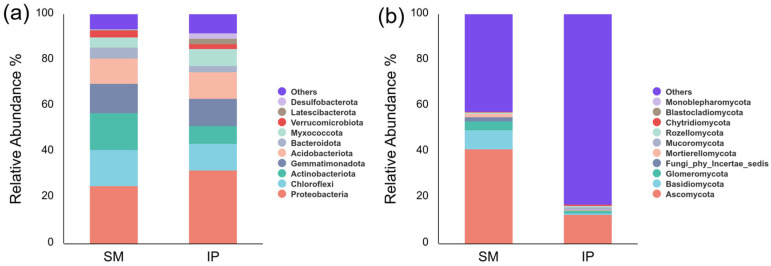
Relative abundances of dominant bacterial (**a**) and fungal (**b**) phyla in soil. SM: sugarcane monoculture; IP: pumpkin intercropping.

A total of 47 bacterial phyla were identified across all soil samples. The predominant phyla in both cropping systems included *Proteobacteria* (25.31–32.20%), *Chloroflexi* (11.51–15.74%), *Actinobacteriota* (7.71–16.22%), *Gemmatimonadota* (11.94–12.72%), *Acidobacteriota* (10.95–11.50%), *Bacteroidota* (2.77–4.91%), *Myxococcota* (4.25–7.52%), and *Verrucomicrobiota* (2.16–2.96%). Collectively, these phyla accounted for 76.82% and 79.61% of the total bacterial sequences in the monoculture and intercropping systems, respectively (Figure 4a). Relative to monoculture, the intercropping system exhibited significantly higher abundances of *Latescibacterota* and *Nitrospirota* and a lower abundance of *Actinobacteriota* (*p* < 0.05). Notably, *Nitrospirota*, a key participant in nitrification and nitrogen cycling, was enriched under intercropping. It rapidly oxidizes toxic nitrite from ammonia-oxidizing bacteria to nitrate, potentially enhancing plant nutrient acquisition [24].

**Figure 5:**
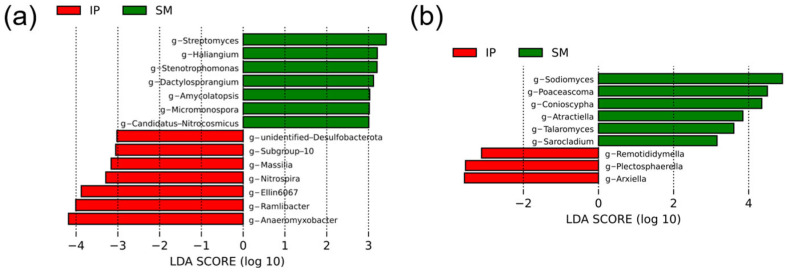
Linear discriminant analysis (LDA) score histograms for differentially abundant bacterial (**a**) and fungal (**b**) genera between sugarcane monoculture (SM) and pumpkin intercropping (IP). A logarithmic LDA score threshold of 3.0 was applied to identify discriminative taxa.

At the genus level, 621 bacterial genera were detected. Linear discriminant analysis (LDA) revealed that genera such as *Anaeromyxobacter*, *Ramlibacter*, *Ellin6067*, and *Nitrospira* were significantly more abundant in the intercropping system, whereas *Streptomyces*, *Haliangium*, *Stenotrophomonas*, and *Dactylosporangium* were more prevalent in monoculture (Figure 5a).

### 3.5. Soil Fungal Community Composition

A total of 14 fungal phyla were identified across all soil samples. The dominant phyla included *Ascomycota* (12.81–41.32%), *Basidiomycota* (0.54–8.41%), *Glomeromycota* (1.08–3.95%), *Fungi_phy_Incertae_sedis* (0.29–1.59%), *Mortierellomycota* (0.23–1.46%), and *Mucoromycota* (0.07–1.23%). These phyla collectively accounted for 56.81% and 16.19% of the total fungal sequences in the monoculture and intercropping systems, respectively (Figure 4b). The relative abundance of *Basidiomycota* was significantly lower in the intercropping system compared to the monoculture (*p* < 0.05).

At the genus level, 340 fungal genera were identified. LDA revealed that *Sodiomyces*, *Poaceascoma*, *Conioscypha*, *Atractiella*, *Talaromyces*, and *Sarocladium* were significantly enriched in the monoculture system, whereas *Rematididymella*, *Piectosphaerella*, and *Arxiella* were more abundant in the intercropping system (Figure 5b).

### 3.6. Relationships Between Soil Microbial Communities and Soil Physiochemical Properties

**Figure 6:**
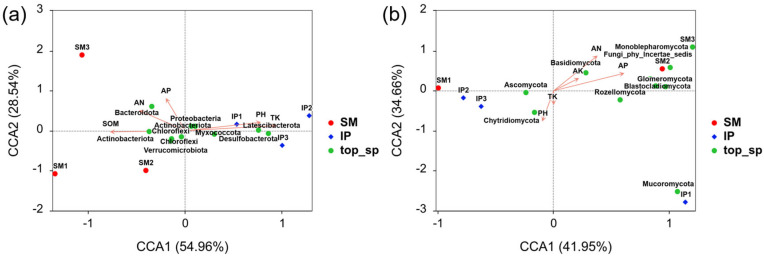
Canonical correlation analysis (CCA) illustrating the relationships between environmental variables and bacterial (**a**) and fungal (**b**) communities under sugarcane monoculture (SM) and pumpkin intercropping (IP) systems.

Canonical correlation analysis (CCA) was used to explore associations between soil physicochemical properties and microbial community structures (Figure 6). For bacterial communities, CCA1 and CCA2 explained 54.96% and 28.54% of the total variance, respectively. TK was significantly correlated with bacterial community composition (r = 0.889, *p* = 0.04). For fungal communities, the first two CCA axes explained 41.94% and 34.70% of the total variance, with AN showing a strong positive correlation (r = 0.898, *p* = 0.007). Taxon–environment correlations revealed that *Proteobacteria*, *Latescibacterota*, *Myxococcota*, and *Desulfobacterota* were positively associated with soil pH and TK, while *Actinobacteriota* showed negative correlations with these variables but was positively associated with SOM. Additionally, *Bacteroidota* showed a positive correlation with AN (Figure 6a). Among fungal taxa, *Blastocladiomycota*, *Mucoromycota*, and *Fungi_phy_Incertae_sedis* were positively associated with AP, whereas *Ascomycota* showed a negative correlation. Additionally, *Basidiomycota* correlated positively with AN, and Chytridiomycota with soil pH (Figure 6b).

## 4. Discussion

Intercropping systems enhance field biodiversity, thereby influencing soil environmental conditions [25]. Several studies have reported that intercropping can increase soil pH3, which aligns with the present findings. Additionally, the sugarcane–pumpkin intercropping system significantly elevated soil TK levels compared to the sugarcane monoculture, consistent with prior research [26,27]. TK is a key indicator of soil fertility, playing a critical role in supporting plant growth and maintaining soil health [28]. Conversely, the intercropping system resulted in a significant reduction in SOM content relative to the monoculture, a finding that contradicts the majority of the existing literature [29,30,31], although a few studies have also observed SOM declines under intercropping conditions [32,33]. This reduction may be attributed to shifts in the microbial populations associated with organic matter decomposition. Furthermore, the decrease in soil organic matter (SOM) content may also be linked to soil nitrogen levels. In the intercropping system, pumpkin’s competition for nitrogen likely triggered enhanced microbial mineralization to accelerate organic matter decomposition, enabling microbes to acquire sufficient nitrogen [34,35]. Lower SOM content can compromise soil aggregate stability, deteriorate soil structure, and impede nutrient cycling processes [36]. Furthermore, soil acidification is known to reduce fertility and restrict root development [37]. Taken together, these findings suggest that sugarcane–pumpkin intercropping improved specific soil quality parameters, thereby partially supporting the first hypothesis.

Soil microbial diversity plays a pivotal role in ecosystem functioning and is essential for sustainable agricultural systems [38,39]. In this study, both alpha and beta diversity indices of bacterial communities were significantly higher under intercropping compared to monoculture, whereas fungal communities exhibited no significant differences (Figure 2 and Figure 3). These results provide only partial support for the second hypothesis. The observed bacterial diversity patterns are consistent with previous studies [3,40,41]. The absence of significant changes in fungal diversity may be due to the inherently longer life cycles of fungi, which could limit the detection of successional changes within the short duration of this study (less than one year) [42,43]. Moreover, the increase in soil pH associated with intercropping may have suppressed the growth of specific fungal taxa sensitive to alkaline conditions [44]. The lack of treatment effects on fungal diversity may also be attributed to either insufficient statistical power in the fungal dataset or amplification bias associated with the ITS primers used [45,46].

Significant shifts in the composition of soil microbial taxa were observed between monoculture and intercropping systems (Figure 4). Notably, the relative abundance of *Actinobacteriota* decreased under intercropping. As a dominant phylum in soil bacterial communities, *Actinobacteriota* plays a pivotal role in the decomposition of organic matter and nutrient cycling [47,48]. Additionally, antibiotics produced by *Actinobacteriota* can influence microbial community dynamics by suppressing the growth of other soil microorganisms [49]. The intercropping system exhibited elevated relative abundances of *Latescibacterota* and *Nitrospirota* compared to the monoculture. The increase in *Nitrospirota*, known for its involvement in nitrification and nitrogen cycling, may enhance plant growth [50]. In contrast, the ecological functions of *Latescibacterota* remain poorly characterized and warrant further investigation. Within the fungal community, the relative abundance of *Basidiomycota* was significantly reduced in the intercropping system. *Basidiomycota* are key decomposers of complex organic compounds such as lignin and cellulose, contributing substantially to organic matter turnover [51,52]. The reduced abundances of *Actinobacteriota* and *Basidiomycota* under intercropping likely contributed to the significantly lower SOM content compared to the monoculture. This interpretation is further supported by LefSe analysis (Figure 5), which showed that genera associated with organic matter decomposition—such as *Streptomyces*, *Haliangium*, *Dactylosporangium*, *Amycolatopsis*, *Talaromyces*, and *Micromonospora*—were enriched in the monoculture system [53,54,55], aligning with the higher SOM levels observed. In contrast, microbial taxa associated with nutrient cycling and plant growth promotion—including *Anaeromyxobacter*, *Nitrospira*, and *Massilia*—were enriched under intercropping [56,57,58]. Notably, *Massilia* has been linked to the suppression of soil-borne pathogens [59,60]. In summary, the shifts in microbial communities within the intercropping system likely contributed to the observed reduction in SOM. However, the intercropping system also exhibited an enrichment of microbial taxa with potential plant growth-promoting and pathogen-suppressing functions. These findings align with our third hypothesis.

Analysis by CCA identified TK as the primary factor driving variation in bacterial community structure, consistent with previous studies [61,62]. Soil potassium content has been correlated with enhanced plant resistance [63], and a significant association between TK levels and *Actinobacteria* abundance, as observed by Wan, was also confirmed in this study [64]. The structure of fungal communities was primarily influenced by AN, in agreement with findings reported by Jiang [65].

## 5. Conclusions

In conclusion, the sugarcane–pumpkin intercropping system regulated key soil physicochemical parameters, specifically pH, TK, and SOM. This cropping pattern enhanced both the alpha and beta diversity of the bacterial community, but had less pronounced effects on fungal communities. Furthermore, the intercropping system facilitated the proliferation of specific bacterial and fungal taxa potentially involved in promoting plant growth and suppressing soil-borne pathogens. Microbial community composition was closely associated with soil properties, with bacterial and fungal assemblages primarily influenced by TK and AN, respectively. The four-month duration of this experiment is relatively short, which likely limits the interpretation of microbial stability or long-term community dynamics. Long-term or multi-seasonal investigations are necessary to evaluate the sustainability impacts of intercropping on microbial succession and soil resilience. We also recommend incorporating functional gene analysis in future studies to further elucidate the functional roles of soil microbial communities.

## Figures and Tables

**Table 1:** Soil physicochemical properties under sugarcane monoculture (SM) and intercropping pumpkin (IP).

Treatments	PH	AN (mg kg^−1^)	AP (mg kg^−1^)	AK (mg kg^−1^)	TK (g kg^−1^)	SOM (g kg^−1^)
SM	6.69 ± 0.16 b	66.89 ± 6.84 a	24.74 ± 6.95 a	58.29 ± 1.51 a	9.82 ± 0.19 b	17.37 ± 0.27 a
IP	7.28 ± 0.08 a	58.64 ± 5.69 a	22.68 ± 2.48 a	59.56 ± 2.49 a	11.58 ± 0.84 a	12.93 ± 1.64 b

Values are presented as mean ± standard deviation. Different letters following the values within the same parameter indicate statistically significant differences (*p* < 0.05; Student’s *t*-test). Each treatment included three biological replicates (n = 3). AN, AP, AK, TK, and SOM denote available nitrogen, available phosphorus, available potassium, total potassium, and soil organic matter, respectively. SM: sugarcane monoculture; IP: intercropping pumpkin.

## Data Availability

Data will be made available on request.
